# Group Sparse Representation Enhances Brain Network Classification of Major Depressive Disorder in Two Chinese Cohorts

**DOI:** 10.31083/AP40685

**Published:** 2026-02-25

**Authors:** Defu Zhang, Cancan Lin, Aoxue Zhang, Xubo Wang, Wenjie Xia, Yue Wang, Yuxin Du, the DIRECT Consortium, Hao Yu, Shanling Ji

**Affiliations:** ^1^School of Mental Health, Jining Medical University, 272000 Jining, Shandong, China; ^2^Department of Psychiatry, Shandong Daizhuang Hospital, 272000 Jining, Shandong, China; ^3^Cheeloo College of Medicine, Shandong University, 250000 Jinan, Shandong, China

**Keywords:** major depressive disorder, sparse representation, support vector machine, classification, brain networks

## Abstract

**Background:**

Major depressive disorder (MDD) is associated with altered organization of functional brain networks. This study aims to evaluate the classification efficacy of three brain networks constructed by Pearson correlation (PC), sparse representation (SR), and group sparse representation (GSR) in distinguishing patients with MDD from healthy controls (HCs).

**Methods:**

The present study involved the recruitment of 117 Chinese participants, comprising 61 individuals diagnosed with MDD and 56 HCs, all of whom underwent functional magnetic resonance imaging (fMRI). Brain time-series signals were extracted from 116 regions to construct whole-brain networks utilizing PC, SR, and GSR. A linear support vector machine (SVM) classifier with least absolute shrinkage and selection operator (LASSO) feature selection was trained using leave-one-out cross-validation (LOOCV) to optimize generalizability. An independent dataset of Chinese (124 first-episode drug-naïve MDD and 105 HCs) was utilized for additional validation.

**Results:**

Compared to the PC and SR, the GSR network yielded superior classification results, with an area under the receiver operating characteristic curve of 0.85, an accuracy of 0.81, and a sensitivity of 0.95. Similar results were observed in the independent MDD dataset. We identified 17 brain connections and 27 brain regions within the GSR network.

**Conclusions:**

Our findings support the adoption of GSR-based brain networks as a robust tool for MDD diagnosis, challenging the conventional reliance on PC in neuroimaging research.

## Main Points

1. The Group Sparse Representation (GSR) method built a brain network that classified Major Depressive Disorder patients significantly better than standard methods, achieving high accuracy and sensitivity.

2. This superior performance was validated in an independent patient dataset, confirming its robustness.

3. The findings establish GSR as a powerful and preferable alternative to the commonly used Pearson Correlation for building diagnostic brain networks in psychiatry research.

## 1. Introduction

Major depressive disorder (MDD) represents a significant contributor to the global psychiatric burden, impacting approximately 300 million individuals worldwide [1]. Over the past decades, substantial progress has been achieved in elucidating the pathophysiological mechanisms underlying MDD [2, 3, 4]. Notably, the brain network has emerged as a prevalent tool for assessing brain functions in individuals with MDD [5, 6, 7].

Brain functional connectivity (FC) between distinct brain regions serves as a primary method for analyzing resting-state functional magnetic resonance imaging (rs-fMRI) data [8, 9, 10]. The construction of a functional brain network typically involves the use of Pearson correlation (PC) [11, 12], which assesses the pairwise linear interactions between different brain regions, with the PC coefficient representing the connectivity weight. Previous studies [13, 14, 15] have extensively documented the application of sparse representation (SR) in various domains such as decomposition, dimensionality reduction, reconstruction [16], face recognition [17], and brain tissue segmentation [18]. In the context of functional brain network research, SR facilitates the construction of sparse networks by evaluating the interrelationships among multiple brain regions at an individual level [14]. The signals from a specific brain region can be represented as a linear combination of signals from other regions, with the corresponding combination weights interpreted as the connections among these regions [14]. The SR method provides enhanced options for constructing brain networks in both healthy individuals [19] and patients with mild cognitive impairment (MCI) [20, 21] and Alzheimer’s disease (AD) [22].

Group sparse representation (GSR) is predominantly employed in the classification of multi-feature, multimodal biometrics [23] and hyperspectral images [24]. In contrast to SR, the GSR network incorporates group constraints and maintains uniformity across all subjects while preserving individual-specific information [25]. The GSR method generates a sparse network through the joint selection or elimination of specific connectivity links applicable to all subjects. Research has demonstrated that the GSR network can be used to effectively classify patients with MCI [25].

To the best of our knowledge, these brain network construction methods have predominantly been employed for the classification of patients with MCI [20, 25, 26] and AD [22]. However, their application in the context of MDD remains inadequately explored. MDD is characterized by significant heterogeneity, state-dependent manifestations, and complex symptomatology, yet it also exhibits certain intrinsic pathological markers whose stability is underrecognized. While neurodegenerative research frequently concentrates on fixed patterns of degeneration, MDD-optimized GSR is capable of capturing both transient state effects and stable trait markers through the application of group constraints. This capability is essential for the study of mood disorders.

The present study sought to evaluate and compare the efficacy of prevalent methodologies in the automatic classification of patients with MDD. A cohort comprising 61 individuals diagnosed with MDD and 56 healthy controls (HCs) was recruited. Brain networks were constructed utilizing PC, SR, and GSR. A linear kernel support vector machine (SVM) classifier was developed to distinguish between MDD patients and HCs. The least absolute shrinkage and selection operator (LASSO) algorithm was employed to select brain connections within each network as features for SVM training. The diagnostic performance was assessed using the leave-one-out cross-validation (LOOCV) approach. The classification performance was quantified through metrics including the area under the receiver operating characteristic (ROC) curve (AUC), accuracy (ACC), sensitivity (SEN), and specificity (SPE). To enhance the validity of our findings, we employed an independent dataset of MDD, sourced from the R-fMRI Maps Project (https://rfmri.org/maps) [27]. We hypothesized that the GSR would surpass alternative methodologies by simultaneously modeling consistent MDD network pathology through the application of $\ell_{2,1}$-norm enforced group sparsity, while also capturing clinically relevant individual variations via subject-specific connection weights [25].

## 2. Methods

### 2.1 Subjects

The study enrolled 61 patients diagnosed with MDD and 56 HCs, all of whom were Chinese, between 18 and 50 years, and who possessed a minimum of seven years of education, and were right-handed (refer to Table 1). The recruitment of MDD patients was conducted through outpatient services from August 2023 to December 2024, while HCs were recruited concurrently via online advertisements. All participants underwent interviews conducted by two experienced psychiatrists utilizing the Structured Clinical Interview for DSM-IV (SCID) [28] and the Mini International Neuropsychiatric Interview (MINI). The severity of depression was evaluated using the 17-item Hamilton Rating Scale for Depression (HAMD-17) [29].

**Table 1.** **Demographic characteristics of the participants in this study**.

	MDD (*n* = 61)	HCs (*n *= 56)	*t*/z/χ^2^	*p* (two-tailed)
Age	35.07 ± 12.55	31.58 ± 9.42	*t* = 1.49	0.14
Gender (male/female)	26/35	31/25	χ^2^ = 1.90	0.17
HAMD	13.98 (7.00, 35.00)	0.69 (0.00, 4.00)	z = 8.41	<0.001

Note: The HAMD scores in the HC group were non-normally distributed, whereas those in the MDD group followed a normal distribution (mean ± standard deviation = 13.98 ± 2.21). Accordingly, HAMD scores for both groups are reported as median (minimum, maximum), and group differences were assessed using the Mann-Whitney U test. HAMD, 17-item Hamilton Rating Scale for Depression; *t*, two-sample test; z, Mann-Whitney U statistic; χ^2^, chi-square test; MDD, major depressive disorder; HCs, healthy controls.

The inclusion criteria for participants with MDD were as follows: (1) age between 18 and 50 years; (2) minimum primary education completion; (3) provision of written informed consent; (4) diagnosis of unipolar MDD episodes following the SCID and MINI; (5) a score of 8 or higher on the HAMD-17; (6) avoidance of all psychotropic medications for a minimum of two weeks prior to study enrollment (four weeks for fluoxetine due to its prolonged half-life). The exclusion criteria for all participants included: (1) a history of epilepsy or brain trauma; (2) severe physical diseases; (3) high risk of suicide; (4) electroconvulsive therapy or transcranial magnetic stimulation within 6 months; (5) pregnancy. In this study, all participants provided written informed consent, and the study protocol received approval from our institutional review board (NO. 202311-HY-1).

### 2.2 MRI Acquisition

All participants underwent scanning using a 3.0 Tesla Siemens Trio MRI scanner (Erlangen, Germany) following specific imaging protocols. Participants were instructed to keep their eyes closed, remain awake and relaxed, and minimize movement during the scan. The resting-state functional MRI (rs-fMRI) parameters were as follows: repetition time (TR)/echo time (TE) = 2000/30 ms, flip angle = 90°, matrix size = 64 × 64, field of view (FOV) = 220 × 220 mm^2^, total of 240 volumes, slice thickness = 3.5 mm, inter-slice gap = 0.6 mm, and number of slices = 33. For the 3D T1-weighted images, the entire brain was covered with 128 sagittal slices, TR = 2000 ms, TE = 3.39 ms, FOV = 256 × 256 mm^2^, flip angle = 7°, slice thickness/gap = 1.33/0 mm, in-plane resolution = 256 × 192 mm, and inversion time (TI) = 1100 ms.

### 2.3 Data Preprocessing

We preprocessed the fMRI data utilizing the Data Processing Assistant for Resting-State fMRI (DPARSF, V4.2, https://rfmri.org/DPARSF) toolbox [30], which operates within the MATLAB (R2022b, https://www.mathworks.com/products/new_products/release2022b.html) environment. The preprocessing steps were as follows: (1) the first ten time points of the rs-fMRI images were discarded due to instability of the initial MRI signal, leaving 230-time points; (2) correction for the acquisition time delay between slices and further realigned to the first volume to correct for head motion incorporating nuisance covariate regression using the Friston 24-parameter model; we reduced respiratory and cardiac effects by using signals from segmentation of the white matter (WM) and cerebrospinal fluid (CSF) compartments in the 3D T1-weighted image as regressors; (3) co-registered T1 structural images to functional images via a nonlinear image registration approach, segmented using a new segment algorithm with diffeomorphic anatomical registration through exponentiated lie algebra (DARTEL); (4) movement parameters for each participant were assessed and participants were excluded if movement exceeded 2 mm or 2° of translation or rotation in any direction; (5) A band-pass frequency filter method (0.01 to 0.08 Hz) was applied to reduce physiological high-frequency noise. The rs-fMRI images were spatially normalized into the Montreal Neurological Institute (MNI) template, and resampled into a spatial resolution of 3 × 3 × 3 mm^3^ and spatially smoothed with a 6 mm full width at half-maximum Gaussian kernel. Subsequently, the resting-state fMRI images were spatially normalized to the MNI template with a resolution of 3 × 3 × 3 mm^3^.

### 2.4 Network Construction

In this study, blood oxygen level-dependent (BOLD) signals were extracted from the entire brain using the Automated Anatomical Labeling (AAL) template, which comprises 116 regions of interest (ROI) [31]. It was assumed that the time series signal X={x⁢(1),x⁢(2),x⁢(3),…,x⁢(n)} represented a single ROI with 230 temporal observations. Detailed information regarding the AAL template is provided in **Supplementary Table 1**.

#### 2.4.1 Pearson Correlation (PC) Network

For any two ROI with time series vectors x and y of length T (time points), the Pearson correlation coefficient *r* was calculated as:

(1) $$r_{x\,y}=\frac{\sum_{t=1}^{T}\left(x_{t}-\bar{x}\right)\,\left(y_{t}-\bar{y}\right)}{\sqrt{\sum_{t=1}^{T}{\left(x_{t}-\bar{x}\right)}^{2}}\,\sqrt{\sum_{t=1}^{T}{\left(y_{t}-\bar{y}\right)}^{2}}}$$

where $x_{t}$ and $y_{t}$ are signal amplitudes at time t, $\bar{x}$ and $\bar{y}$ are mean signal values, and T is the total number of time points (after preprocessing). All coefficients were converted to z-scores using Fisher’s r-to-z transformation to ensure normality. The transformed z-scores were assembled into a 116 × 116 symmetric connectivity matrix for each subject, with diagonal elements set to zero.

#### 2.4.2 Sparse Representation (SR) Network

In contrast to the PC method, the SR brain network was constructed utilizing linear regression. Brain signals from the *r*th ROI $x_{i}^{r}$were regressed by the signals from all the other ROI $X_{i}^{r}$using an *l*_1_-norm sparse regularization [15] as Eqn. (2):

(2) $$W_{i}^{r}=\arg\min_{W_{i}^{r}}\frac{1}{2}\,{∥x_{i}^{r}-X_{i}^{r}\,W_{i}^{r}∥}_{2}^{2}+\lambda \,{∥W_{i}^{r}∥}_{1}$$

where λ controls the sparsity of $W_{i}^{r}$. Finally, non-zero weights were Fisher z-transformed for normality, and symmetric 116 × 116 connectivity matrices were constructed by averaging bidirectional connections (diagonals set to zero). This yielded sparser networks than PC.

#### 2.4.3 Group Sparse Representation (GSR) Network

Based on the SR method, the GSR network was constructed by using *l*_2,1_-norm regularization across all subjects within-group [15] as Eqn. (3):

(3) $$W^{r}=\arg\min_{W^{r}}\,\sum_{i=1}^{N}\left(\frac{1}{2}\,{∥X_{i}^{r}-X_{i}^{r}\,W_{i}^{r}∥}_{2}^{2}\right)+\lambda \,{∥W^{r}∥}_{2,1}$$

where $W^{r}=\left[w_{1}^{r},w_{2}^{r},w_{3}^{r},\ldots \,\ldots \,w_{N}^{r}\right]$ represents the *r*th ROI of all subjects, and N is the number of subjects in a group. Non-zero weights underwent Fisher z-transformation before constructing symmetric 116 × 116 connectivity matrices through bidirectional averaging (diagonal = 0), yielding networks that were sparser than PC while more reproducible than SR.

### 2.5 Feature Extraction and Selection

The connection coefficients calculated using the PC, SR, and GSR methods were extracted as features to represent network properties. To minimize the feature set, we employed the widely adopted LASSO technique for feature selection in the training of the SVM classifier.

### 2.6 Classifier Training and Performance Evaluation

In this study, a linear kernel SVM was employed to evaluate the discriminative capability of features extracted from three distinct network methodologies. Feature selection was conducted using the LASSO. The regularization parameter (λ) for LASSO was optimized within the range of 0.01 to 0.1, with increments of 0.01, as determined by previous neuroimaging research that has demonstrated this range to yield optimal feature selection performance [15, 32]. To address the challenge of limited sample size in our sample, we implemented a nested LOOCV scheme to build optimal SVM models and obtain an unbiased estimate of generalization classification performance. Specifically, for N subjects in the study, one subject was excluded for testing, while the remaining N-1 subjects were used to construct the optimal SVM model. From these N-1 subjects, N-1 distinct training subsets were created by sequentially excluding one additional sample, resulting in N-2 subjects in each training subset. Functional connectivity construction, feature extraction, and feature selection were performed for each subset. The performance of each combination of SVM parameters and selected features was evaluated using the second excluded subject. The combination yielding the best performance was then used to build the optimal SVM model for future classification. This procedure was repeated N-1 times, each time with a different training subset. For classification of a completely novel test sample, all N-1 classifiers were used, and the final classification decision was made through majority voting. This process was iterated N times, each time omitting a different subject, resulting in an overall cross-validation classification accuracy. Additionally, the optimal value of lambda in the SR and GSR equations was identified through a grid search methodology. Concurrently, we calculated the true positive (TP), false positive (FP), false negative (FN), and true negative (TN) rates in each iteration. The classification performance of the three methods was evaluated using ACC, SEN, and SPE, which were defined as follows:

(4) $$\mathrm{ACC}\left(\%\right)=\frac{T\,P+T\,N}{T\,P+T\,N+F\,P+F\,N}\times 100\%$$

(5) $$\mathrm{SEN}\left(\%\right)=\frac{T\,P}{T\,P+F\,N}\times 100\%$$

(6) $$\mathrm{SPE}\left(\%\right)=\frac{T\,N}{T\,N+F\,P}\times 100\%$$

We used an independent dataset of Chinese individuals diagnosed with MDD from the R-fMRI Maps Project (rfmri.org/maps) [27] to further substantiate our findings. In this study, we included a total of 229 participants, comprising 124 unmedicated patients experiencing a drug-naïve first episode of MDD and 105 HCs. This independent validation cohort with both similarities and distinctions versus our primary cohort, and matched Chinese ethnicity, and age range. Both used SCID/MINI diagnosis, and HAMD. The demographic details of the participants in this publicly available dataset are presented in **Supplementary Table 2**. The preprocessing of fMRI data and the analysis of classification performance were conducted using the same methodologies as applied to our own MDD sample set.

### 2.7 Statistical Analysis

All continuous demographic variables (age, HAMD-17 scores) were assessed for normality using the Shapiro-Wilk test (α = 0.05) and for homogeneity of variance using Levene’s test. If the assumptions of normality (*p* < 0.05) or equal variance were violated, non-parametric alternatives (Mann-Whitney U test) were applied. For normally distributed data, two-sample *t*-tests were used to compare group differences. Group differences in sex distribution were assessed using the chi-square (χ^2^) test.

## 3. Results

### 3.1 Performance Comparison

**Fig. 1.**
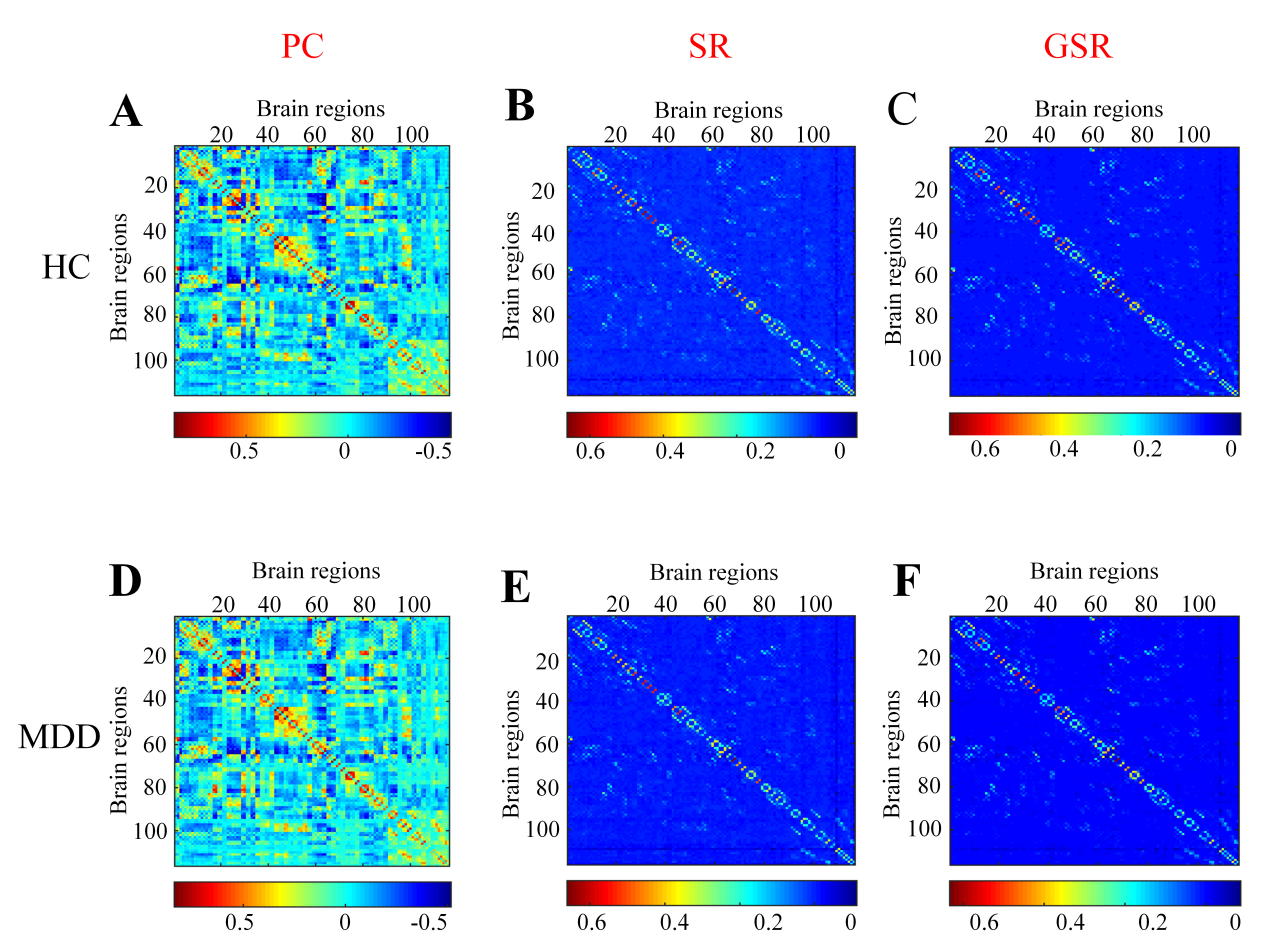
**The functional brain networks derived using the Pearson correlation (PC), sparse representation (SR), and group sparse representation (GSR) methods for HCs (A–C) and MDD (D–F)**.

Functional brain networks constructed via PC, SR, and GSR exhibited distinct topological patterns between MDD patients and HCs (Fig. 1A–F). The detailed numerical values corresponding to these six connectivity patterns displayed in Fig. 1A–F are shown in the **Supplementary Excel Files**.

**Fig. 2.**
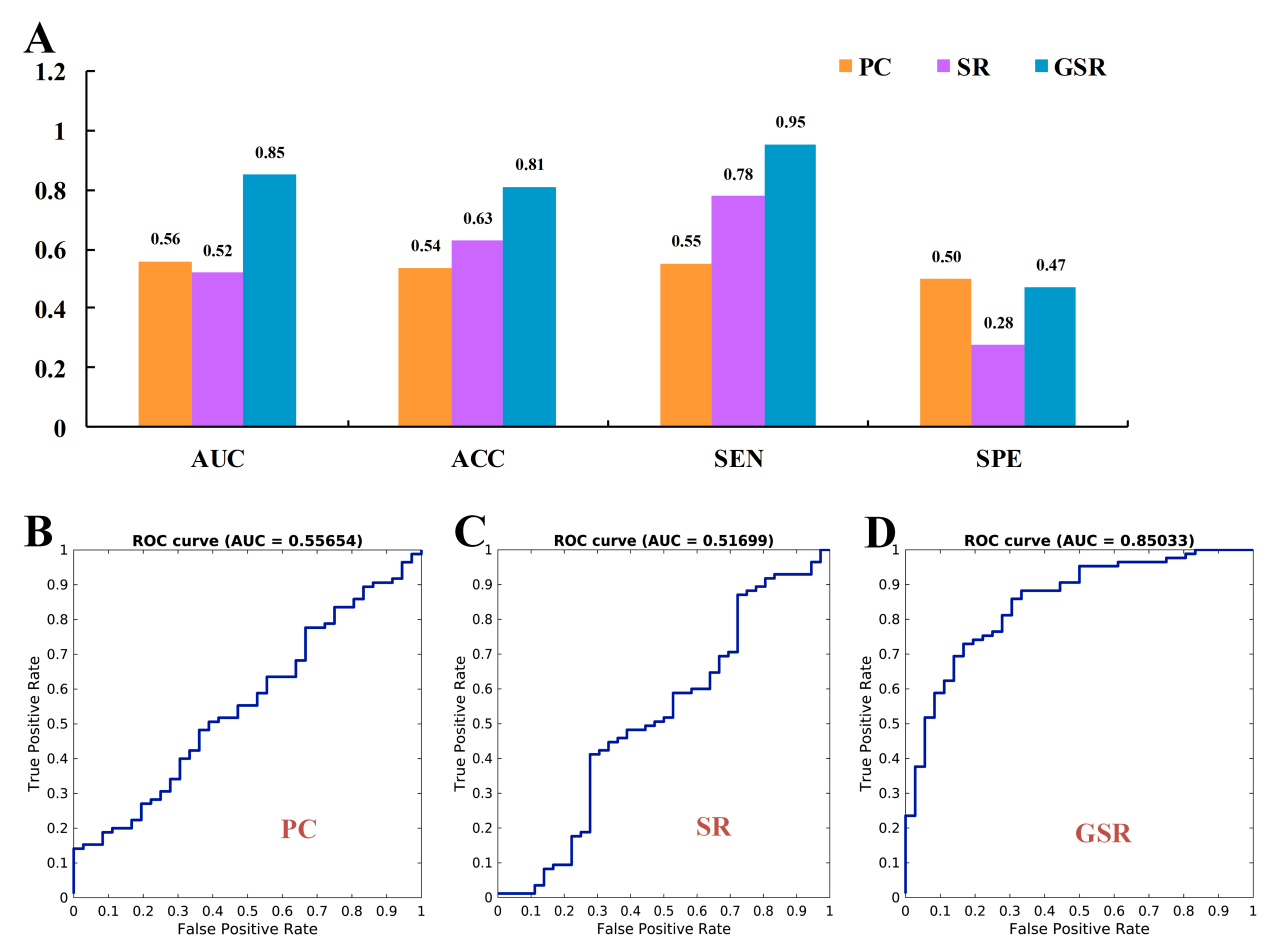
**Classification performance metrics for the Pearson correlation (PC), sparse representation (SR), and group sparse representation (GSR) methods**. (A) presents the area under the curve (AUC), accuracy (ACC), sensitivity (SEN), and specificity (SPE). (B–D) display the receiver operating characteristic (ROC) curves for PC, SR, and GSR, respectively.

Fig. 2 demonstrates that the GSR method attained the highest values for AUC, ACC, and SEN, with 0.85, 0.81, and 0.95, respectively. In contrast, the PC network exhibited the lowest ACC and SEN, with values of 0.54 and 0.55, and achieved the highest SPE with 0.50. The SR method recorded the lowest AUC and SPE, with values of 0.52 and 0.28, respectively.

In the independent validation cohort, the GSR method demonstrated superior performance, achieving the highest values for AUC, ACC, SEN, and SPE, with respective values of 0.65, 0.66, 0.72, and 0.59. In contrast, the PC network recorded the lowest performance metrics, with AUC, ACC, SEN, and SPE values of 0.50, 0.46, 0.20, and 0.50, respectively. The SR method exhibited moderate performance, with AUC, ACC, SEN, and SPE values of 0.60, 0.59, 0.64, and 0.54, respectively. See **Supplementary Fig. 1**.

### 3.2 Classification Performance of GSR network

**Fig. 3.**
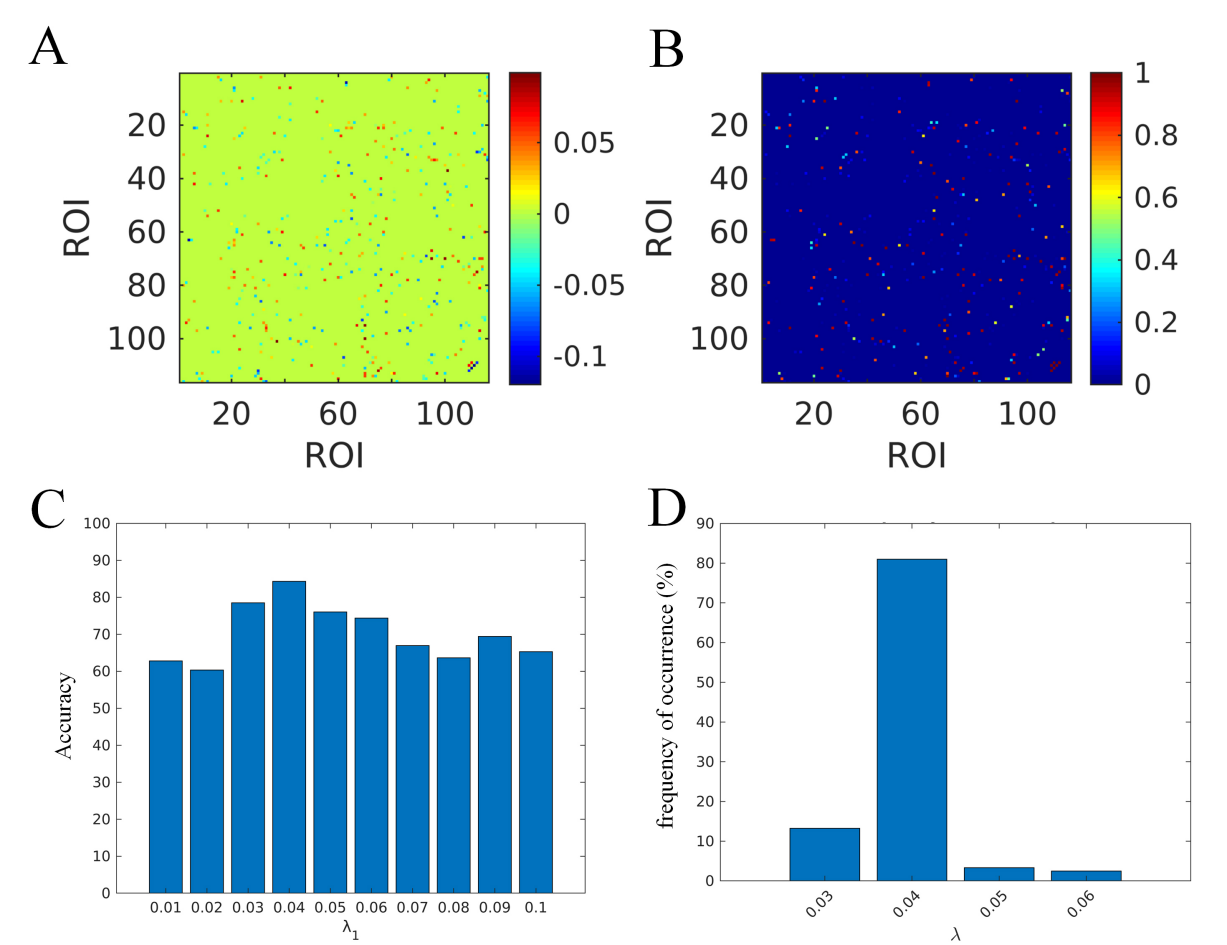
**The following elements pertain to the optimal classification model for GSR**. (A) the mean weight assigned to each connection; (B) the normalized frequency of each connection’s occurrence; (C) the classification accuracy associated with each lambda value; (D) the frequency of occurrence for each lambda value. ROI, regions of interest.

Fig. 3A,B illustrates the most discriminative brain connections within the GSR network as identified by the SVM classifier. The mean weight assigned to each connection and the normalized frequency of each connection’s occurrence are shown in **Supplementary Excel Files**. The GSR network achieved a peak accuracy of 0.81 (Fig. 3C) and demonstrated the highest frequency of occurrence in the model robustness evaluation (Fig. 3D) when the regularization parameter λ was set to 0.04 in the SVM model. The results of PC and SR are provided in the **Supplementary Figs. 2,3**.

The performance comparison results and optimal models for these three methods, as applied to the public dataset, are presented in the **Supplementary Figs. 1,4,5,6**). The GSR method demonstrated superior performance within this dataset, as illustrated in **Supplementary Fig. 1**.

### 3.3 Most Discriminative Brain Regions and Connections in the GSR Network

Table 2 provides a summary of the 17 discriminative brain connections and 27 brain regions identified within the GSR network, as determined by the weighting coefficients of the SVM analysis. These 27 brain regions comprise 14 cortical regions, 3 subcortical regions, and 10 cerebellar areas. These connections and regions demonstrate that both cortical and cerebellar regions participate in local connections (within cortical or cerebellar regions) as well as global connections (extending beyond their respective regions to interact with other regions). The subcortical regions exhibit two connections with cerebellar regions and one connection with the cortical regions.

**Table 2.** **The most discriminative brain connections and regions in the GSR network**.

Connection number	ROI1		ROI2	
	Index	Brain region names	Index	Brain region names
1	24	Right superior frontal gyrus, medial	11	Left inferior frontal gyrus, opercular part
2	64	Right supramarginal gyrus	35	Left posterior cingulate gyrus
3	65	Left angular gyrus	38	Right hippocampus
4	66	Right angular gyrus	47	Left lingual gyrus
5	65	Left angular gyrus	55	Left fusiform gyrus
6	86	Right middle temporal gyrus	78	Right thalamus
7	95	Left cerebellum.3	67	Left precuneus
8	93	Left cerebellum.Crus2	69	Left paracentral lobule
9	95	Left cerebellum.3	70	Right paracentral lobule
10	96	Right cerebellum.3	11	Left inferior frontal gyrus, opercular part
11	100	Right cerebellum.6	70	Right paracentral lobule
12	105	Left cerebellum.9	71	Left caudate nucleus
13	112	Vermis.6	75	Left lenticular nucleus, pallidum
14	112	Vermis.6	30	Right insula
15	112	Vermis.6	109	Vermis.1.2
16	114	Vermis.8	70	Right paracentral lobule
17	111	Vermis.4.5	110	Vermis.3

Note: In the same row, a connection exists between ROI1 and ROI2. The regions of interest (ROI) were labeled according to the AAL116 template, with both the indices and the names of the brain regions referenced from this template.

## 4. Discussion

The present study investigated the classification performance of brain networks which were constructed using PC, SR, and GSR in Chinese patients diagnosed with MDD. The results indicated that GSR achieved the highest classification performance, suggesting that brain connections that incorporate both inter-subject variability and within-group similarity may be more effective in identifying patients with MDD. This conclusion was further confirmed by an independent dataset of Chinese MDD patients. The most discriminative brain connections were identified between the cerebral cortex and the cerebellum, suggesting their pivotal role in distinguishing MDD patients from HCs and challenging the traditional limbic-centric model of depression pathophysiology. The findings of this study make significant contributions to advancing the field of MDD identification and provide additional evidence regarding the classification efficacy of sparse brain networks.

### 4.1 The Performances of Three Networks

The PC network has been extensively investigated in the context of various neurological disorders [33]. The PC method was the predominant technique employed in neuroimaging research [34, 35]. This correlation method cannot reveal the interaction effects among several brain regions [34]. In the present study, the SR network, which incorporated inter-regional brain effects into its network construction, demonstrated superior classification performance compared to the PC method. Moreover, the GSR network, which integrates both individual and group-level information into the SR network, achieved the highest classification performance. These findings are consistent with previous research on patients with MCI [20, 25, 26] and AD [22].

The SR network exhibited increased inter-subject variability, resulting in distinct network topological structures for individual subjects [36]. This variability could potentially impair generalization ability due to the heterogeneity or inconsistency across subjects [25, 36], despite achieving superior classification performance compared to PC. In contrast, the GSR method sparsely represented brain connections at the group level, simultaneously enforcing intrinsic local sparsity and nonlocal self-similarity within a unified framework. The brain network derived from GSR encompassed both inter-subject variability and within-group similarity [37], thereby contributing to optimal classification performance in MDD patients.

The GSR approach exhibits heightened sensitivity to network abnormalities specific to MDD in both datasets, potentially due to its distinctive capability to model concurrently. Specifically, the *l*_2,1_-norm regularization enforces group-wise sparsity by selectively preserving connections that consistently exhibit alterations across MDD patients, while simultaneously eliminating noisy individual variations. Furthermore, GSR maintains group consistency while allowing for subject-specific weight adjustments for preserved connections [15, 38], thereby accommodating the clinical heterogeneity inherent in MDD. This dual capacity of GSR to identify both shared biomarkers and personalized variants renders it particularly well-suited for addressing the complex pathophysiology of MDD, where population-level abnormalities coexist with clinically significant heterogeneity.

The relatively low SPE observed across methods (PC: 0.50, SR: 0.28, GSR: 0.47) highlights two critical considerations. First, the extreme sparsity of SR may lead to the exclusion of essential negative controls that are present in the denser PC networks, which also led to the lowest AUC value of 0.52. In contrast, GSR constraints more effectively preserve these discriminative null connections. Second, the notably low SEN and AUC of SR are consistent with evidence indicating that subtypes of MDD exhibit divergent patterns [39, 40, 41], resulting in the misclassification of true negatives when employing individual-level sparse networks. Additionally, LASSO’s tendency to favor positive correlations may lead to a disproportionate selection of connections that exhibit MDD hyperconnectivity. However, this bias was mitigated in GSR through the use of the $\ell_{2,1}$-norm, which ensures group consistency. These findings underscore that while sensitivity effectively captures connectivity changes associated with the disease, achieving high specificity remains challenging in the classification of MDD. This difficulty likely reflects the complex, multi-network pathophysiology of the disorder.

### 4.2 Neurobiological Significance of Identified Connections in the Pathophysiology of MDD

The discriminative connections identified through GSR network analysis align with established neuropathological findings in MDD, elucidating a coherent framework of circuit-level dysfunction [42, 43]. The left inferior frontal gyrus (IFG), recognized as a hub for cognitive control and regulation of emotion [44, 45, 46], exhibits altered connectivity patterns that directly contribute to core depressive symptomatology [47, 48, 49]. Its impaired connections with both the right superior frontal gyrus and the cerebellum indicate: (1) disrupted top-down cognitive control, as evidenced by deficits in executive function; (2) abnormal emotional processing, manifesting as a negative bias; and (3) dysregulated monoaminergic modulation, supported by cerebellar-prefrontal neurotransmitter pathways observed in animal studies [50, 51, 52, 53]. This dysfunction within the prefrontal-cerebellar circuit operates in conjunction with abnormalities in the limbic system, where alterations in angular gyrus-hippocampus connectivity are associated with maladaptive memory processes, such as overgeneralized negative recall [54, 55]. Furthermore, insula-vermis dysregulation underlines characteristic somatic symptoms, including fatigue and altered pain perception. The involvement of bilateral paracentral lobules further indicates potential deficits in sensorimotor integration, which may contribute to psychomotor disturbances observed in MDD [56]. These network abnormalities collectively impair higher-order executive functioning through three primary mechanisms: (1) cognitive-emotional integration failure, where prefrontal-cerebellar dysconnectivity disrupts the capacity for regulation of emotion; (2) memory processing bias, in which limbic-cerebellar abnormalities promote a preferential recall of negative experiences; and (3) sensorimotor dysynchronization, where the involvement of the paracentral lobes alters bodily perception and motor response. Future research should explore whether these connectivity patterns exhibit differential sensitivity to specific treatment modalities, such as repetitive transcranial magnetic stimulation targeting prefrontal versus cerebellar nodes.

It should be noted that the limbic system is frequently identified as an atypical brain region in individuals with MDD [57, 58, 59]. The absence of limbic connections (e.g., amygdala-hippocampus-prefrontal pathways) in our GSR reflects the critical GSR’s group-consistency requirement ($\ell_{2,1}$-norm) and may suppress highly variable limbic connections. The identified cortico-cerebellar connections may represent more reliable biomarkers because they are less contaminated by state-dependent mood fluctuations and align with emerging cerebellar roles in the regulation of emotion. Nevertheless, the relative absence of limbic system connections warrants further investigation. Future studies should examine state-dependent and trait effects and test whether combining cortico-cerebellar and limbic connectivity improves diagnostic specificity.

### 4.3 Limitations

Several methodological limitations should be acknowledged in this study. First, our analysis was restricted to undirected functional connectivity networks, which, while computationally efficient, may not fully capture the directional information flow between brain regions that could provide greater neurobiological insight. Future research should more effectively incorporate connectivity methods [60] and alternative network construction approaches (partial correlation, graphical LASSO, mutual information) to better characterize the complex, potentially directional interactions in MDD. Second, while this study employed three methods for network construction, there are additional methodologies worth considering, such as the strength and similarity-guided GSR approach [15]. The primary objective of this study was to evaluate the classification performance of methods commonly utilized in brain network research within a cohort of individuals with MDD. Consequently, recently proposed methods that have not been widely adopted were not included. Moreover, Sparse methods (SR/GSR) enhance interpretability by emphasizing dominant neural connections. However, the sparsity induced by the choice of the regularization parameter (λ) introduces significant neurobiological trade-offs. Excessive sparsity may lead to the omission of subtle yet functionally critical pathways. The selection of λ is crucial, as higher values (>0.04) tend to disproportionately eliminate long-range connections, whereas lower values (<0.04) may retain excessive noise. This sensitivity to λ highlights the advantage of GSR’s balanced approach (λ = 0.04), which surpasses SR by achieving greater biomarker stability without compromising biological plausibility. Notably, dense methods such as PC maintain complementary value by capturing features of network resilience and state-dependent plasticity that lie below sparsity thresholds. Future research should integrate GSR’s robust identification of core circuits with PC’s sensitivity to weaker connections within these subnetworks, while validating findings against multimodal evidence, such as lesion studies and dynamic causal modeling. This hybrid strategy may provide a more comprehensive understanding of complex network pathology in MDD, which encompasses both strong and subtle connectivity alterations. Third, although the present study concentrated on LASSO due to its demonstrated effectiveness in handling high-dimensional neuroimaging data, we recognize the potential advantages of conducting a more extensive comparative analysis. Future research should systematically assess hybrid feature selection pipelines, such as the integration of SVM-RFE with LASSO, alongside advanced classifiers, including ensemble methods like Gradient Boosting Decision Tree, to enhance discriminative capabilities. Such comparative analyses would necessitate larger datasets to ensure robust generalizability. Fourth, this study did not investigate the correlations between brain networks and patient behaviors or genotypes, which are associated with MDD symptoms, such as the serotonin transporter gene [61, 62]. Future research should aim to determine whether the most discriminative connections exhibit stronger correlations with these factors.

## 5. Conclusions

In this study, we evaluated the classification performance of functional brain networks constructed using PC, SR, and GSR in patients with MDD. Utilizing the SVM algorithm, our results demonstrated that the GSR approach yielded superior classification performance. This finding suggests that brain networks that integrate individual connectivity information into a group framework may be more effective for identifying MDD patients. Our results imply that while PC-based networks are prevalent in numerous studies, they may not represent the optimal method. This research contributes additional evidence regarding the efficacy of the classification of sparse brain networks.

## Availability of Data and Materials

The datasets generated and analyzed during the current study are available from the corresponding author on reasonable request.

## Supplementary Material

Supplementary material associated with this article can be found, in the online version, at https://doi.org/10.31083/AP40685.
