# Supplementary material for: Group Sparse Representation Enhances Brain Network Classification of Major Depressive Disorder in Two Chinese Cohorts
Source: Alpha Psychiatry. 2026 Feb 25;27(1):40685. doi: 10.31083/AP40685 (PMC12957975; doi:10.31083/AP40685)
Supplement: Supplementary file 1 [file 2757-8038-27-1-40685-s1.zip › Supplementary Material.docx]

**Supplementary Materials**

**Data Preprocessing**

(1) discarded the first ten time points of the rs-fMRI images due to instability of the initial MRI signal, leaving 230-time point; (2) corrected for the acquisition time delay between slices and further realigned to the first volume to correct for head motion incorporating nuisance covariate regression using the Friston 24-parameter model; Besides, we reduced respiratory and cardiac effects by using signals from segmentation of the white matter (WM) and cerebrospinal fluid (CSF) compartments in the 3D T1-weighted image as regressors; (3) coregistered T1 structural images to functional images via a nonlinear image registration approach and segmented using a new segment algorithm with diffeomorphic anatomical registration through exponentiated lie algebra (DARTEL); (4) assessed movement parameters for each participant and participants were excluded if movement exceeded 2 mm or 2°of translation or rotation in any direction; (5) A band-pass frequency filter method (0.01 to 0.08 Hz) was applied to reduce physiological high-frequency noise. Finally, the rs-fMRI images were spatially normalized into the Montreal Neurological Institute (MNI) template, and resampled into a spatial resolution of 3×3×3 mm³ and spatially smoothened with a 6 mm full width at half-maximum Gaussian kernel.

**Supplementary Table**

(page: 2-4)

**Supplementary Table 1**. Brain region labels in the AAL 116 template

| number | Label | number | Label | number | Label | number | Label | number | Label |
| --- | --- | --- | --- | --- | --- | --- | --- | --- | --- |
| 1 | Precentral_L | 25 | Frontal_Med_Orb_L | 49 | Occipital_Sup_L | 73 | Putamen_L | 97 | Cerebelum_4_5_L |
| 2 | Precentral_R | 26 | Frontal_Med_Orb_R | 50 | Occipital_Sup_R | 74 | Putamen_R | 98 | Cerebelum_4_5_R |
| 3 | Frontal_Sup_L | 27 | Rectus_L | 51 | Occipital_Mid_L | 75 | Pallidum_L | 99 | Cerebelum_6_L |
| 4 | Frontal_Sup_R | 28 | Rectus_R | 52 | Occipital_Mid_R | 76 | Pallidum_R | 100 | Cerebelum_6_R |
| 5 | Frontal_Sup_Orb_L | 29 | Insula_L | 53 | Occipital_Inf_L | 77 | Thalamus_L | 101 | Cerebelum_7b_L |
| 6 | Frontal_Sup_Orb_R | 30 | Insula_R | 54 | Occipital_Inf_R | 78 | Thalamus_R | 102 | Cerebelum_7b_R |
| 7 | Frontal_Mid_L | 31 | Cingulum_Ant_L | 55 | Fusiform_L | 79 | Heschl_L | 103 | Cerebelum_8_L |
| 8 | Frontal_Mid_R | 32 | Cingulum_Ant_R | 56 | Fusiform_R | 80 | Heschl_R | 104 | Cerebelum_8_R |
| 9 | Frontal_Mid_Orb_L | 33 | Cingulum_Mid_L | 57 | Postcentral_L | 81 | Temporal_Sup_L | 105 | Cerebelum_9_L |
| 10 | Frontal_Mid_Orb_R | 34 | Cingulum_Mid_R | 58 | Postcentral_R | 82 | Temporal_Sup_R | 106 | Cerebelum_9_R |
| 11 | Frontal_Inf_Oper_L | 35 | Cingulum_Post_L | 59 | Parietal_Sup_L | 83 | Temporal_Pole_Sup_L | 107 | Cerebelum_10_L |
| 12 | Frontal_Inf_Oper_R | 36 | Cingulum_Post_R | 60 | Parietal_Sup_R | 84 | Temporal_Pole_Sup_R | 108 | Cerebelum_10_R |
| 13 | Frontal_Inf_Tri_L | 37 | Hippocampus_L | 61 | Parietal_Inf_L | 85 | Temporal_Mid_L | 109 | Vermis_1_2 |
| 14 | Frontal_Inf_Tri_R | 38 | Hippocampus_R | 62 | Parietal_Inf_R | 86 | Temporal_Mid_R | 110 | Vermis_3 |
| 15 | Frontal_Inf_Orb_L | 39 | ParaHippocampal_L | 63 | SupraMarginal_L | 87 | Temporal_Pole_Mid_L | 111 | Vermis_4_5 |
| 16 | Frontal_Inf_Orb_R | 40 | ParaHippocampal_R | 64 | SupraMarginal_R | 88 | Temporal_Pole_Mid_R | 112 | Vermis_6 |
| 17 | Rolandic_Oper_L | 41 | Amygdala_L | 65 | Angular_L | 89 | Temporal_Inf_L | 113 | Vermis_7 |
| 18 | Rolandic_Oper_R | 42 | Amygdala_R | 66 | Angular_R | 90 | Temporal_Inf_R | 114 | Vermis_8 |
| 19 | Supp_Motor_Area_L | 43 | Calcarine_L | 67 | Precuneus_L | 91 | Cerebelum_Crus1_L | 115 | Vermis_9 |
| 20 | Supp_Motor_Area_R | 44 | Calcarine_R | 68 | Precuneus_R | 92 | Cerebelum_Crus1_R | 116 | Vermis_10 |
| 21 | Olfactory_L | 45 | Cuneus_L | 69 | Paracentral_Lobule_L | 93 | Cerebelum_Crus2_L |  |  |
| 22 | Olfactory_R | 46 | Cuneus_R | 70 | Paracentral_Lobule_R | 94 | Cerebelum_Crus2_R |  |  |
| 23 | Frontal_Sup_Medial_L | 47 | Lingual_L | 71 | Caudate_L | 95 | Cerebelum_3_L |  |  |
| 24 | Frontal_Sup_Medial_R | 48 | Lingual_R | 72 | Caudate_R | 96 | Cerebelum_3_R |  |  |

**Supplementary Table 2.** Demographic and clinical variables in HCs and MDD patients.

|  | MDD (n = 124) | HC (n = 105) | t /χ^2^ | *p* value (two-tailed) |
| --- | --- | --- | --- | --- |
| Age | 29.89 ± 8.30 | 28.71 ± 7.93 | t = 1.09 | 0.227 |
| Gender | 43 / 81 | 50 / 55 | χ^2^ = 3.95 | 0.047 |
| Education | 12.86 ± 3.29 | 13.58 ± 2.31 | t = -1.89 | 0.061 |
| HAMD | 18.63 ± 10.46 | - |  |  |

Note: Data are presented as the mean ± SD. The *p*-value was obtained using two samples t-test.

Abbreviations: HAMD = 17-item Hamilton Rating Scale for Depression; SD = standard deviation; t = two samples t-test.

**Supplementary Figures**

(page: 5-10)


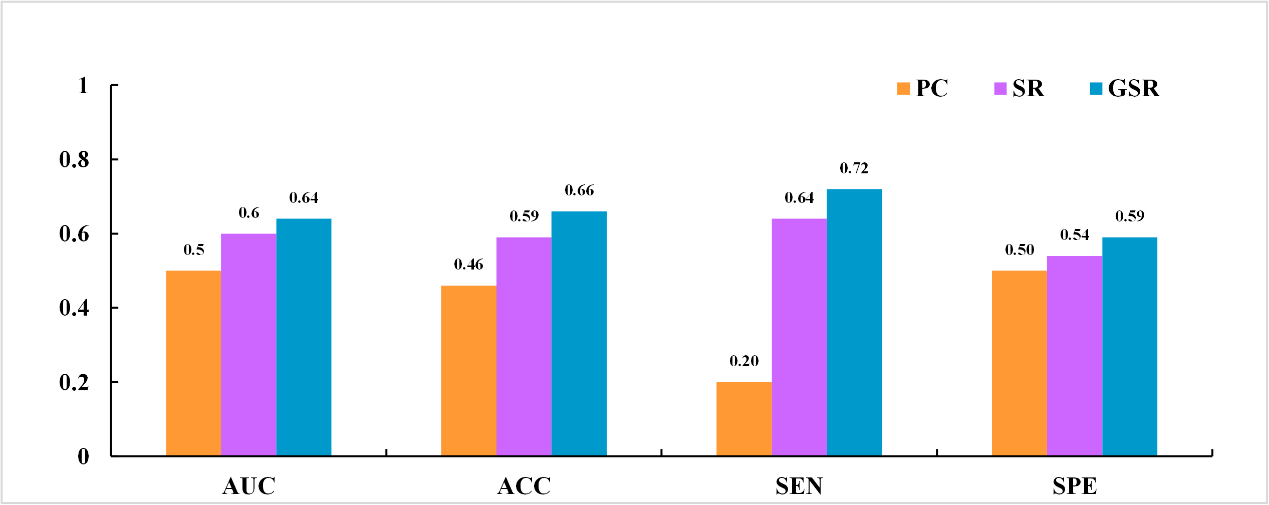


**Supplementary Fig. 1**. Classification performances of the PC, SR, and GSR.

PC= Pearson correlation; SR = sparse representation; GSR = group sparse representation; ACC = accuracy, SEN = sensitivity; SPE = specificity.


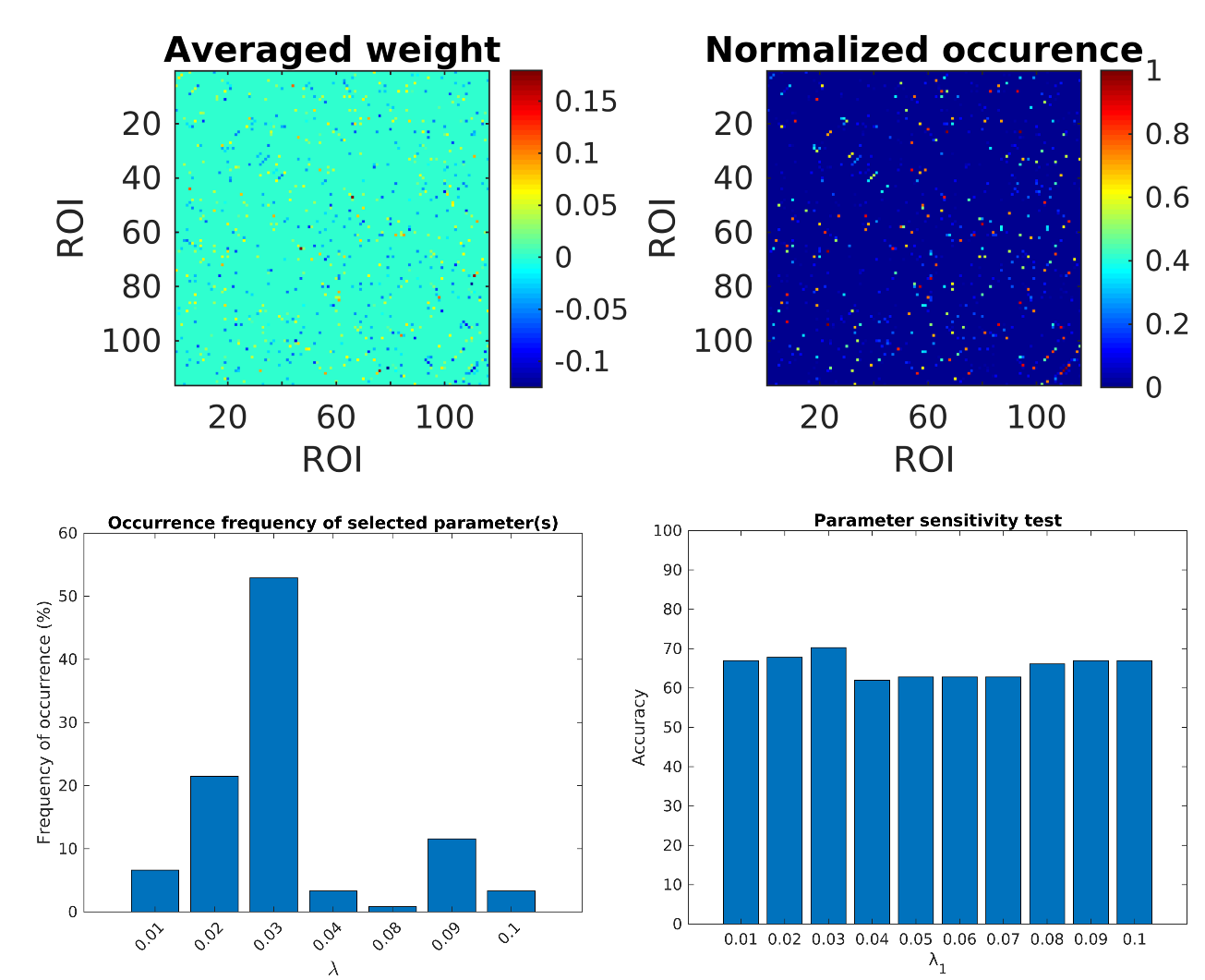


**Supplementary Fig. 2**. The connections and parameters of the optimum classification model for sparse representation (SR) network.


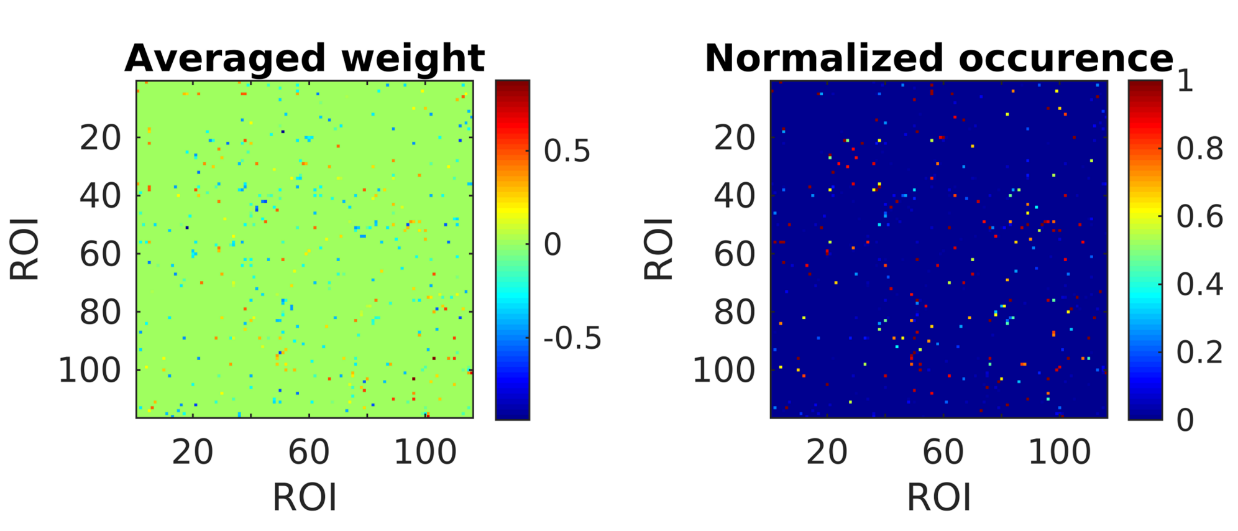


**Supplementary Fig. 3**. The connections of the optimum classification model for Pearson correlation (PC) network.


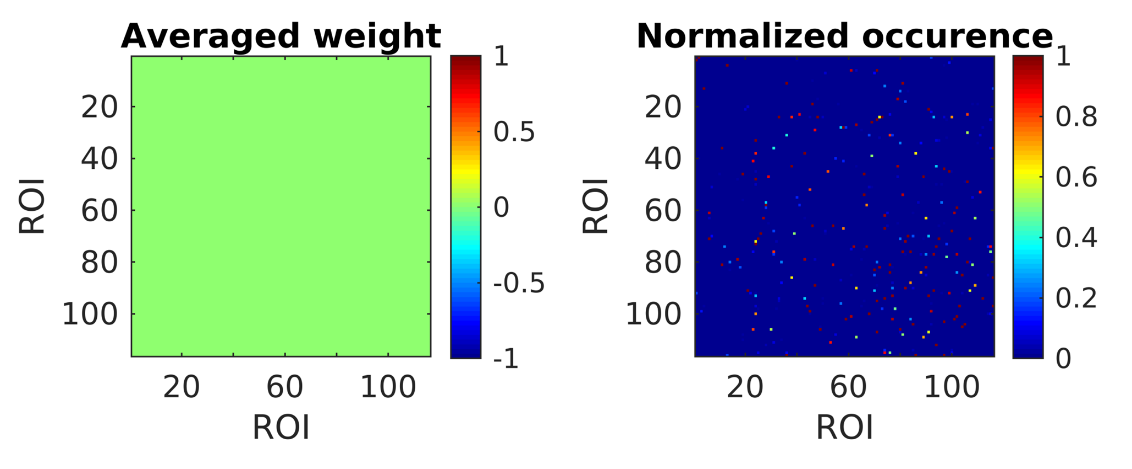


**Supplementary Fig. 4**. The connections of the optimum classification model for Pearson correlation (PC) network.


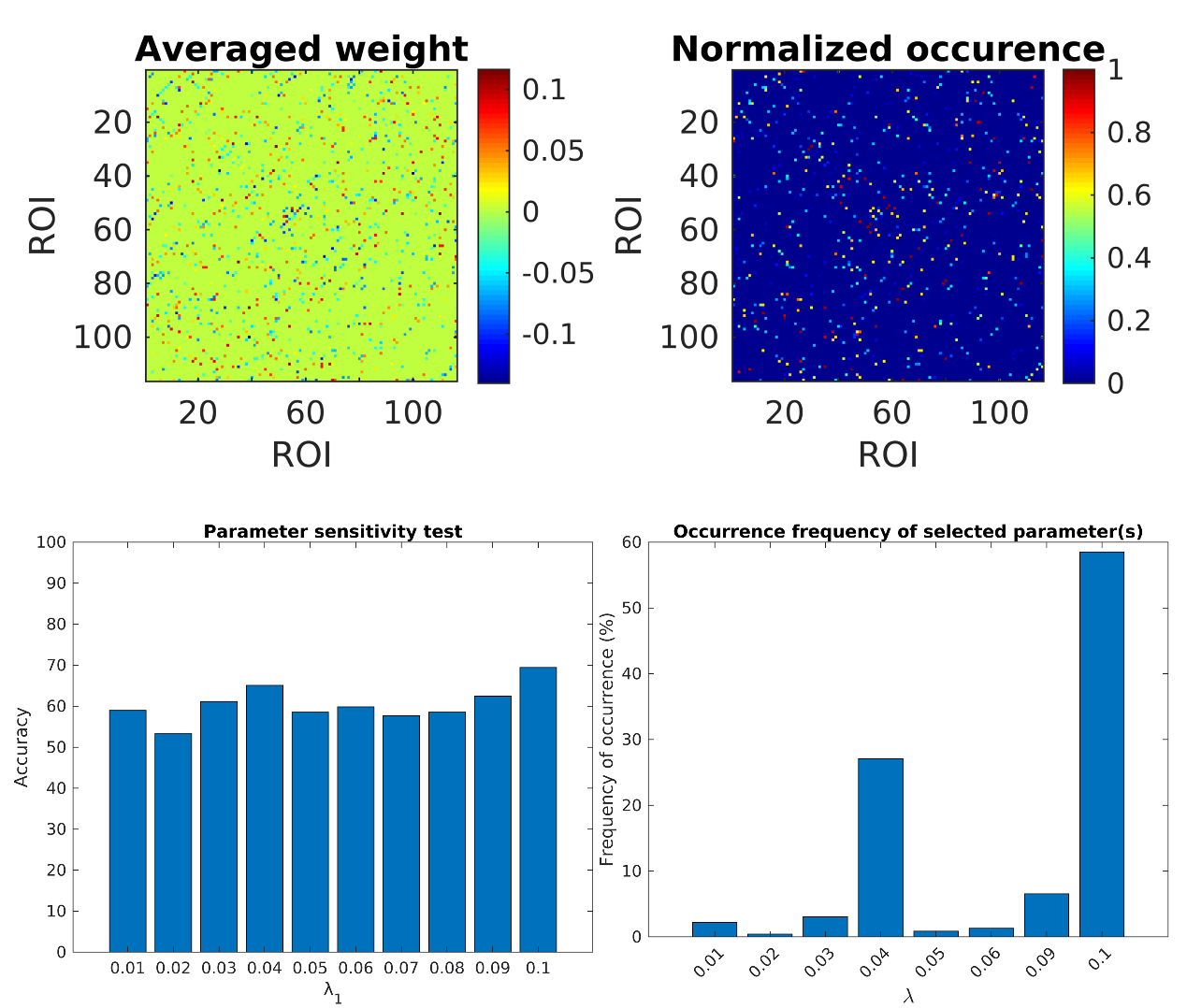


**Supplementary Fig. 5**. The connections and parameters of the optimum classification model for sparse representation (SR) network.


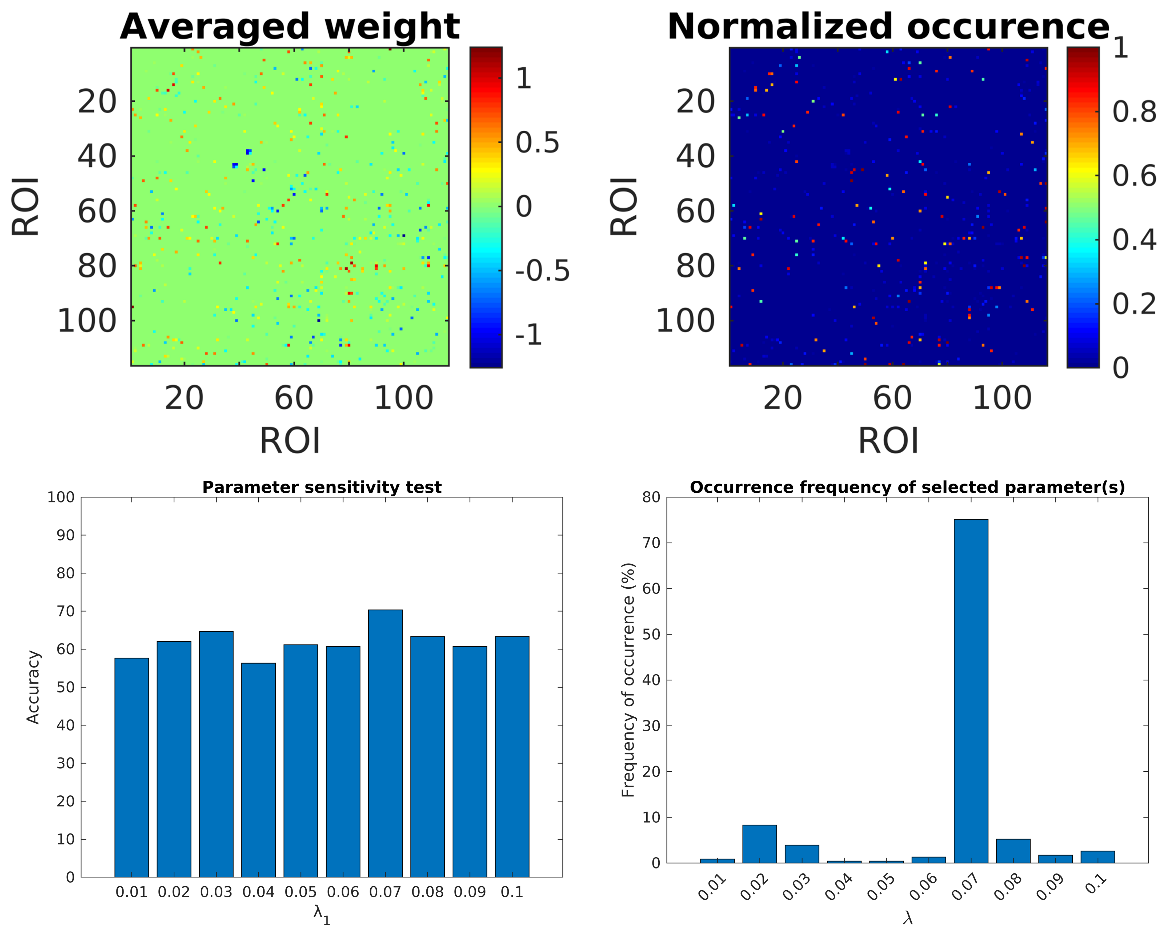


**Supplementary Fig. 6**. The connections and parameters of the optimum classification model for group sparse representation (GSR) network.
